# Sézary Syndrome: Different Erythroderma Morphological Features with Proposal for a Clinical Score System

**DOI:** 10.3390/cells11030333

**Published:** 2022-01-20

**Authors:** Gabriele Roccuzzo, Silvia Giordano, Gianluca Avallone, Marco Rubatto, Silvia Canonico, Ada Funaro, Erika Ortolan, Rebecca Senetta, Paolo Fava, Maria Teresa Fierro, Simone Ribero, Pietro Quaglino

**Affiliations:** 1 Section of Dermatology, Department of Medical Sciences, University of Turin, 10126 Torino, Italy; silvia.giordano94@gmail.com (S.G.); gianluca.avallone@hotmail.it (G.A.); rubattomarco@gmail.com (M.R.); silvia.canonico23@gmail.com (S.C.); fava_paolo@yahoo.it (P.F.); mariateresa.fierro@unito.it (M.T.F.); simone.ribero@unito.it (S.R.); pietro.quaglino@unito.it (P.Q.); 2 Laboratory of Immunogenetics, Department of Medical Sciences, University of Turin, 10126 Torino, Italy; ada.funaro@unito.it (A.F.); erika.ortolan@unito.it (E.O.); 3 Pathology Unit, Department of Oncology, University of Turin, 10126 Torino, Italy; rebecca.senetta@unito.it

**Keywords:** Sézary syndrome, cutaneous lymphoma, CTCL, mycosis fungoides, erythrodermic

## Abstract

Sézary syndrome is a rare subtype of cutaneous T-cell lymphoma characterized by erythroderma, peripheral lymphadenopathies, and circulating atypical cerebriform T-cells. To date, no definite staging system has been developed for these patients. In this retrospective analysis of the archive of the Dermatological Clinic of the University of Turin, Italy, erythrodermic SS patients were classified according to clinical records and photographs into three main presentations: erythematous, infiltrated, or melanodermic. The pattern of erythroderma was found to be associated with disease outcome, as better survivals were recorded in patients with erythematous and infiltrative erythroderma. Patients in the melanodermic group, though less represented in our investigation, seemed to show a worse trend in survival. According to this preliminary evidence, a new prognostic classification, with a revised score specific for Sézary syndrome patients, can be proposed to usefully integrate the current staging system. The correlation displayed in our research will be hopefully confirmed by prospective studies with larger cohorts, with the aim of identifying significant prognostic features in this subset of cutaneous T-cell lymphoma patients.

## 1. Introduction

Sézary syndrome (SS) represents the leukemic and erythrodermic type of cutaneous T-cell lymphoma (CTCL) [1]. It is traditionally defined by the triad of pruritic erythroderma, generalized lymphadenopathy, and clonally related neoplastic T cells with cerebriform nuclei (Sézary cells) in the skin, lymph nodes, and peripheral blood. It accounts for 2% of all CTCL and carries a poor prognosis, with a reported 5-year disease-specific survival of 36% [2]. The presence of high blood involvement (B score = 2, according to the TNMB classification) is a diagnostic criterion for SS and is defined by different parameters, such as in addition to the demonstration of clonally related neoplastic T cells in the skin and peripheral blood, either an absolute Sézary cell (SC) count of >1000/µL, or an expanded CD4+ T-cell population resulting in a CD4/CD8 ratio ≥ 10, CD4+/CD7− cells ≥ 30%, or CD4+/CD26− cells ≥ 40% are required [3]. A recent consensus by the European Organization for Research and Treatment of Cancer (EORTC) has suggested defining blood involvement based on phenotypical, rather than morphological, criteria in the presence of more than 1000/mm^3^ CD4+ CD26− or CD4+ CD7− cells [4]. From a clinical point of view, erythroderma represents the most relevant parameter in SS patients, even though early cases can present without erythrodermic features [5,6]. Nevertheless, the T parameter of the TNMB staging system is not useful in distinguishing the possible clinical manifestations in these patients, as they are all classified as T4 regardless of different clinical features. Moreover, this parameter does not allow the evaluation of prognostic differences in terms of distinct SS phenotypes. To date, no other definite skin staging system has been developed for SS patients. In this single centre retrospective study, our pluri-decennial database on SS patients has been reviewed. The objectives were: (1) to characterize erythroderma morphological features at diagnosis and to evaluate the modifications during time; (2) to correlate erythroderma patterns with the disease evolution and survival; (3) to suggest a cutaneous score to stage these patients, related to a different clinical course.

## 2. Materials and Methods

**Figure 1:**
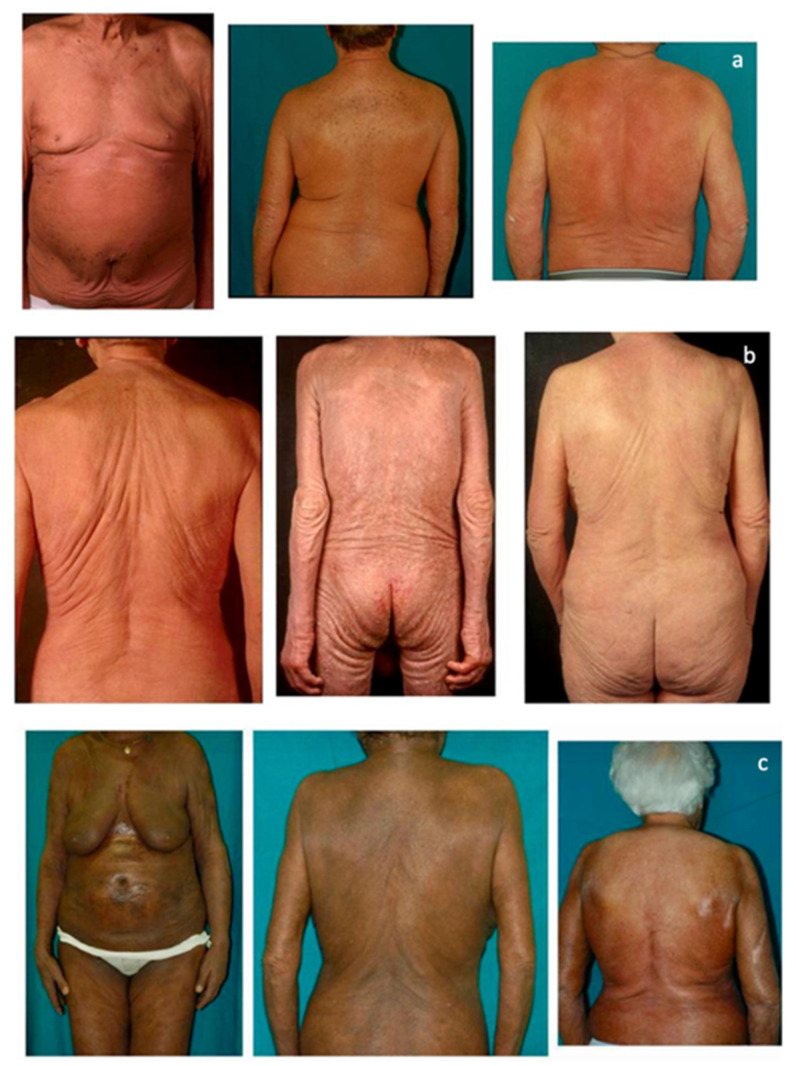
(**a**) The erythematous presentation (**b**) The infiltrative presentation (**c**) The melanodermic presentation.

This retrospective observational single centre study was carried out at the Dermatology Clinic of the University of Turin, an Italian tertiary referral centre on CTCLs. The database of the clinic was reviewed and all cases with a confirmed SS diagnosis, according to international criteria, between January 1977 and December 2019 were included. Inclusion criteria were: availability of assessment of circulating SC at diagnosis, availability of the clinical picture at diagnosis, and a more than 6-month follow-up. A total of 159 patients had demographic and laboratory/phenotype data; however, 15 of them lacked clinical pictures at diagnosis and/or clinical data as to disease evolution and follow-up. A total of 144 patients were therefore included in our research. All clinical pictures available in the database were reviewed in a blinded way by two authors (P.Q., MT.F.). According to literature data, clinical experience, and careful clinical picture analysis, a consensus was reached to consider three different patterns of clinical erythroderma: erythematous, infiltrative, and melanoderma. Erythematous erythroderma was defined as widespread reddening, with or without exfoliation, affecting at least 80% of body surface area. The distinction between erythematous and infiltrative was based on the presence of skin folds and more evident thickness in the infiltrative cases. Melanoderma was defined as a widespread darkening affecting at least 80% of the body surface area. Patients without a clear-cut erythroderma, showing confluent patches and/or plaques or sub-erythroderma, were considered as a distinct group. Representative pictures of each erythrodermic group are displayed in Figure 1. The evaluation of circulating Sézary cells, as well as diagnostic procedures and imaging, was related to the time of diagnosis. Circulating Sézary cells were determined using morphological features, then incorporated with flow-cytometry considering cluster of differentiation (CD) expression [7,8,9,10]. Survival rates have been recorded and median survival rates among the different subtypes have been compared through the log-rank statistical test using GraphPad Prism software.

## 3. Results

Patients’ characteristics at diagnosis are summarized in Table 1.

### 3.1. Clinical Characteristics of Patients

There was a male prevalence (61.1% vs. 38.9%), with a median age at diagnosis of 70 years (range 25–97). About one-third of patients (27.8%) had received a previous diagnosis of Mycosis Fungoides. The overall median SC count at diagnosis was 2691 (range 147–52,419); 15.20% of patients presented with an SC count > 10.000/µL, whereas the majority (63.9%) presented with mid-range values (1.000–10.000/µL) and the remaining 20.9% had a count < 1.000/µL. All cases with a value of circulating SC lower than 1000/mm^3^ showed a rapid increase to more than 1000/mm^3^ during follow-up. As for characteristic clinical features, 24.5% of the patients had ectropion, 61.5% showed palmoplantar keratoderma, 36.4% alopecia, and 11.9% facies leonine. Nodular lesions in association with erythroderma were found in 17.5% of patients at diagnosis or during follow-up. Remarkably, 59% of patients developed infectious events during follow-up, with cutaneous infection as the most common type.

### 3.2. Erythroderma Morphological Features

**Figure 2:**
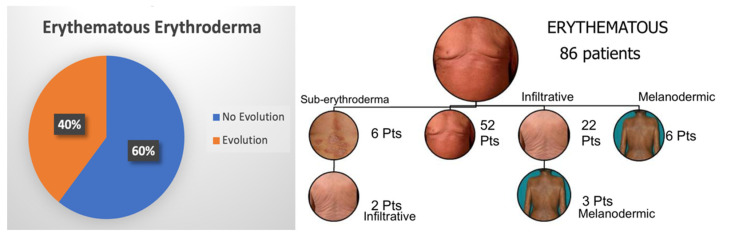
Percentage of erythematous erythrodermic patients who showed a clinical downgrading or evolution to infiltrative/melanodermic patterns.

**Figure 3:**
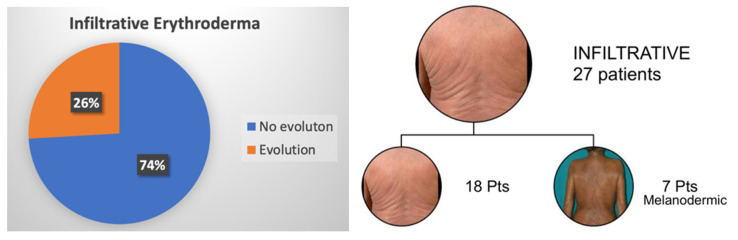
Percentage of infiltrative erythrodermic patients who showed a clinical evolution to melanoderma.

At the time of diagnosis, 122 patients (84.7%) showed erythroderma, whilst 22 (15.3%) had confluent patches/plaques/nodules or sub-erythroderma and therefore were not included in the analysis. As for the former group, most patients showed an erythematous pattern (86 patients), followed by the infiltrative (27 patients) and the melanodermic variants (9 patients). Pattern evolution was assessed for each patient. In the erythematous subgroup, 34 patients (40%) developed a morphological evolution, and more commonly, the infiltrative pattern (70% of cases). Only a few patients (5%) showed a downgrading of manifestations (i.e., from erythrodermic to sub-erythrodermic), secondary to response to therapy. As for the infiltrative pattern, all 7 patients (26%) who developed an evolution showed the progression to melanoderma. On the contrary, no melanodermic patient showed a downgrading of erythroderma. Remarkably, melanodermic patients showed a higher number of circulating Sezary cells compared to the other patterns. Figure 2 and Figure 3 summarize the percentage of observed erythrodermic evolution in the two main subgroups, with the specific pattern evolutions assessed in our study.

**Figure 4:**
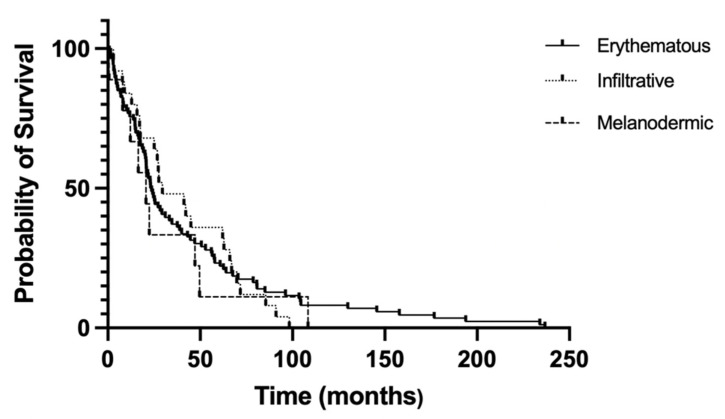
Overall survival in months according to erythrodermic subtype.

Median survival rates were analyzed for each patient. The erythematous erythrodermic group showed a median survival of 23.4 months (1.95 years). The infiltrative erythrodermic group displayed a median survival of 29.5 months (2.46 years). As for melanodermic patients, a lower median survival of 20.6 months (1.72 years) was recorded, even though the survival analysis did not reach statistical significance. Landmark analysis showed that patients with erythematous erythroderma had better long-term survivals, as only patients in this group survived beyond the 104-month landmark point. Figure 4 reports the survival curves of the three erythrodermic groups.

## 4. Discussion

SS is a rare form of leukemic manifestation of CTCL. Still today, overall survival rates are low, varying from 7.5 to 22.4 months, and very few therapeutic options have proved to be effective [11]. Treatment choice depends on multiple factors, including disease extension; for patients with low tumor burden, recommendations provide extracorporeal photopheresis (ECP) as a first-line therapy, which can be used alone or in combination with other agents such as retinoids/bexarotene, and interferon-alpha, alone or combined with skin-directed therapy/retinoids [12,13]. For more advanced stages, with high blood tumor burden, it is preferable to start with debulking agents and then use ECP; in this scenario anti-CCR4 mogamulizumab can induce a high response rate, particularly in the blood, thus reducing the number of atypical cells. Other monoclonal antibodies, such as anti-CD52 alemtuzumab and anti-CD30 brentuximab have also shown significant clinical activity in patients with advanced SS [14]. Traditionally, these patients have been treated with chemotherapy, (i.e., gemcitabine, liposomal doxorubicine, CHOP, and CHOP-like polychemotherapy), but considering the low response and the high toxicity, it should not be taken into account as a primary treatment [15]. To date, allogeneic hematopoietic stem cell transplantation (HSTC), particularly using reduced-intensity conditioning, is the only curative treatment, although intended for only few selected patients [16,17,18]. Overall, SS patients have an unmet clinical need for effective treatments, due to low response rates, short-lived improvements, concomitant immunosuppression, and often severe drug-related side effects [19]. While the current staging system draws a distinction between early and advanced mycosis fungoides stages, no factor considers the difference in clinical SS manifestations—in fact, all SS patients, regardless of their erythrodermic appearance, are staged as T4 B2. The rationale of this preliminary study has been to address the potential correlation between different SS erythrodermic features and prognosis. In our retrospective observational study, the most represented subtypes were the erythematous (59.7%) and infiltrative (18.8%) erythroderma patterns. Interestingly, 40% of patients in the former group and 26% of patients in the latter showed a clinical appreciable evolution, while no patients with melanoderma showed a downgrading of erythroderma intensity. The survival analysis seems to suggest that different erythrodermic manifestations may be related to different prognoses. In fact, erythematous erythrodermic and infiltrative erythrodermic patients seem to have better median survival (23.4 and 29.5 months, respectively), while patients with melanodermic patterns and high blood tumor burden tend to display worse prognosis (median survival rate of 20.6 months). More importantly, a landmark analysis of long-term survival shows that patients with erythematous erythroderma have longer survivals. A limitation of our study is represented by the exiguous number of melanodermic patients, making studies with larger cohorts much-needed to confirm the emerging trend displayed in this study between erythroderma subtypes and survival. Overall, based on these results, we suggest that the following clinical parameters could be taken into account in order to refine SS stage definitions from a clinical point of view. SS erythrodermic patients could be therefore classified according to three different clinical morphological phenotypes, i.e., erythrodermic (E1), infiltrative (E2), melanodermic (E3), with progressively worse long-term survival. Table 2.

Moreover, this classification may reflect different histologic findings. To date, only a few studies have investigated the histopathologic features of melanodermic SS and hemosiderosis has been described as a possible origin of hyperpigmented SS [20]. Overall, the most important discriminating histologic feature in SS is the presence of a monotonous bandlike or perivascular infiltrate in the papillary dermis, mainly composed of large cerebriform-mononuclear cells. The presence of intraepidermal nests of atypical cells (Pautrier's microabscesses) can also be observed [21,22,23]. Since among these erythrodermic classes (E1, E2, E3) there is a progressive increase in skin fold thickness, we expect on histology a progressively increased perivascular infiltrate, from a few lymphocytes in erythematous erythroderma, to more conspicuous infiltrates in infiltrative forms and frank hemosiderosis in melanoderma [24]. The limit of this classification relies on the difficulties in differentiating the erythroderma morphotypes (particularly erythematous and infiltrative). Therefore, this proposed classification needs to be validated in prospective studies that could verify its reproducibility and clinical feasibility in larger multicenter cohorts, possibly integrating former tools, such as the Modified Severity-Weighted Assessment Tool (mSWAT), with the abovementioned pigmentation features [25]. Future studies could be also focused on the interpretation of the different pathological substrate and phenotypical features of circulating atypical cells [26] associated with these different clinical morphological features, as well as to identify whether a different therapeutic approach could be applied to better tailor these different morphological subtypes.

## Figures and Tables

**Table 1:** Patients’ characteristics at diagnosis.

Parameters at Diagnosis	N° Patients	Erythematous (E1)	Infiltrative (E2)	Melanoderma (E3)	Confluent Patches/Plaques/ Sub-Erythroderma
N° patients	144	86	27	9	22
Gender M-F (% Male patients)	88–56 (61.1%)	51–35 (59.3%)	22–5 (81.5%)	5–4 (55.5%)	10–12 (45.5%)
Age (years) (median; range)	70 (25–97)	70 (25–97)	69 (50–84)	73 (55–86)	67 (49–93)
				
Previous MF	40 (27.8%)	23/86 (26.7%)	8/27 (29.6%)	1/9 (11.1%)	8/22 (36.4%)
Circulating SC (median; range)(/mm^3^)	2691 (147–52,419)	3228 (147–44,153)	1698 (212–13,349)	4398 (420–52,419)	3160 (261–50,730)

**Table 2:** New staging proposal according to SS features.

SS Subtype	Proposed Staging	Clinical Presentation	Features
Patches, Plaques, Sub-erythroderma	E0	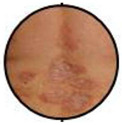	No frank erythroderma. It may characterize early as well as atypical forms of SS.
Erythematous Erythroderma	E1	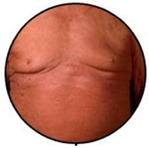	Widespread reddening, with or without exfoliation, affecting at least 80% of body surface area. No appreciable signs of increased skin fold thickness.
Infiltrative Erythroderma	E2	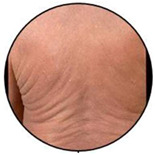	Widespread reddening, with or without exfoliation, affecting at least 80% of body surface area and clear features of increased skin fold thickness.
Melanoderma	E3	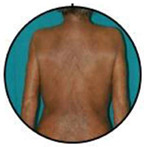	Widespread darkening affecting at least 80% body surface area.

## Data Availability

The data presented in this study are available on request from the corresponding author. The data are not publicly available due to privacy reasons.
